# Electronic self-administered screening for substance use in adult primary care patients: feasibility and acceptability of the tobacco, alcohol, prescription medication, and other substance use (myTAPS) screening tool

**DOI:** 10.1186/s13722-019-0167-z

**Published:** 2019-10-15

**Authors:** Angéline Adam, Robert P. Schwartz, Li-Tzy Wu, Geetha Subramaniam, Eugene Laska, Gaurav Sharma, Saima Mili, Jennifer McNeely

**Affiliations:** 1 Department of Population Health, New York University (NYU) School of Medicine, 180 Madison Avenue, 17th floor, New York, NY 10016 USA; 2 Friends Research Institute, 1040 Park Avenue, Suite 103, Inc, Baltimore, MD 21201 USA; 3 Department of Psychiatry and Behavioral Sciences, Department of Medicine and Duke Clinical Research Institute, Duke University Medical Center, Durham, NC 27710 USA; 4 Center for the Clinical Trials Network, National Institute on Drug Abuse, National Institutes of Health, 6001 Executive Boulevard, Bethesda, MD 20892 USA; 5 Department of Psychiatry, NYU Langone School of Medicine, New York, NY 10016 USA; 6 The EMMES Corporation, 401 North Washington Street, Rockville, MD 20850 USA

**Keywords:** Substance use disorders, Drug abuse screening, Alcohol screening, Alcohol abuse, Primary care, Acceptability of health care, Self-administered screening, Electronic screening

## Abstract

**Background:**

The TAPS Tool is a substance use screening and brief assessment instrument that was developed for use in primary care medical settings. It is one of the first screening instruments to provide rapid assessment of all commonly used substance classes, including illicit and prescription opioids, and is one of the only available screeners designed and validated in an electronic self-administered format (myTAPS). This secondary analysis of data from the TAPS Tool validation study describes the feasibility and acceptability of the myTAPS among primary care patients.

**Methods:**

Adult patients (N = 2000) from five primary care clinics completed the TAPS Tool on a tablet computer (myTAPS), and in an interviewer-administered format. Requests for assistance and time required were tracked, and participants completed a survey on ease of use, utilization of audio guidance, and format preference. Logistic regression was used to examine outcomes in defined subpopulations, including groups that may have greater difficulty completing an electronic screener, and those that may prefer an electronic self-administered approach.

**Results:**

Almost all participants (98.3%) reported that the myTAPS was easy to use. The median time to complete myTAPS screening was 4.0 min (mean 4.48, standard deviation 2.57). More time was required by participants who were older, Hispanic, Black, or reported non-medical prescription drug use, while less time was required by women. Assistance was requested by 25% of participants, and was more frequently requested by those who with lower education (OR = 2.08, 95% CI 1.62–2.67) or age > 65 years (OR = 2.79, 95% CI 1.98–3.93). Audio guidance was utilized by 18.3%, and was more frequently utilized by participants with lower education (OR = 2.01, 95% CI 1.54–2.63), age > 65 years (OR = 1.79, 95% CI 1.22–2.61), or Black race (OR = 1.30, 95% 1.01–1.68). The myTAPS format was preferred by women (OR = 1.29, 95% CI 1.00–1.66) and individuals with drug use (OR = 1.43, 95% CI 1.09–1.88), while participants with lower education preferred the interviewer-administered format (OR = 2.75, 95% CI 2.00–3.78).

**Conclusions:**

Overall, myTAPS screening was feasible and well accepted by adult primary care patients. Clinics adopting electronic screening should be prepared to offer assistance to some patients, particularly those who are older or less educated, and should have the capacity to use an interviewer-administered approach when required.

## Background

Tobacco, alcohol and drug use drive poor health outcomes and are associated with substantial societal costs [1–5]. The World Health Organization (WHO), and the U.S. Preventive Service Task Force (USPSTF) recommend screening for tobacco and alcohol use in adult primary care patients [5–7]. Screening for drug use is recommended in the US Surgeon General’s report on addiction, and by the Substance Abuse and Mental Health Services Administration (SAMHSA) [8, 9]. Screening for opioid use has gained increased attention in light of the US opioid crisis, as individuals who are identified as having problem use of opioids could be targeted for overdose prevention and treatment interventions. New SAMHSA guidelines specifically recommend screening for opioid use in general medical settings [10]. Doing so requires the use of a screening tool that identifies illicit and prescription opioid use as a component of a general screen for problem use of tobacco, alcohol, and other drugs, while still remaining brief enough to fit into routine clinical care.

Despite the existing recommendations, unhealthy alcohol and drug use remain largely undetected in health care settings [1, 11]. Many challenges to implementing screening are related to clinical workflow and time pressures [12, 13], as well as to the stigma associated with substance use [14, 15]. An electronic self-administered screening tool has the potential to address several of these barriers that are encountered in medical settings. First, patients may feel more comfortable disclosing stigmatized behavior when it is self-reported instead of asked face-to-face, and this can lead to more accurate disclosure of substance use [16–18]. Further, electronic screening may allow patients to complete screening in the privacy of their own home, (for example through a patient portal into their electronic health record (EHR)), and be linked immediately to a patient-facing electronic intervention that does not require them to interact with clinical staff. Second, electronic screening tools can reduce barriers to screening in medical settings because they can be completed in the clinic waiting room using a tablet or kiosk computer, with results transmitted directly into the EHR, thus minimizing intrusion into clinical workflows. Screening results may be paired with clinical decision supports tools in the EHR, to help providers offer adequate interventions to their patients (i.e. brief intervention for unhealthy use or treatment for substance use disorder). Third, an electronic approach can improve the quality of screening. Because self-administered questionnaires consistently deliver screening items exactly as written, electronic screening may have higher fidelity and reliability than an interviewer-administered approach [19–23]. Delivering screening in an electronic format, as opposed to paper, also makes it possible to deliver sophisticated instruments that may require complex skip patterns or computer adaptive testing approaches, (for example, the World Health Organization Alcohol, Smoking, and Substance Involvement Screening Test (ASSIST), or the Patient-Reported Outcomes Measurement Information System (PROMIS) instruments) because the computer efficiently delivers only the items that are required based on the patient’s prior responses [23–25].

Yet electronic screening could also be challenging. Patients may have trouble reading or understanding the questions, or difficulty navigating the computer interface. While electronic self-administered screening tools can incorporate audio guidance to accommodate low literacy users, some patients may still have difficulty operating the technology [21, 26–28]. While some patients appreciate the privacy of self-administered screening, others may prefer the human touch of an interviewer [29].

The tobacco, alcohol, prescription medication, and other substance use (TAPS) Tool is a two-step screening (TAPS-1) and brief assessment (TAPS-2) instrument [30, 31] that identifies unhealthy use of tobacco, alcohol, prescription medications (used nonmedically) and illicit drugs. The TAPS Tool was specifically developed for adult primary care, was designed to be used in either an electronic self-administered format (myTAPS) or a more traditional interviewer-administered format, and both formats were validated in a large study conducted by the National Institute of Drug Abuse (NIDA) Clinical Trials Network [30, 31]. The TAPS Tool performed well for identifying problem use of tobacco (sensitivity 0.92, specificity 0.87), alcohol (sensitivity 0.77, specificity 0.77), and commonly used classes of illicit drugs (sensitivity ranging from 0.73 to 0.79, specificity ranging from 0.93 to 1.0) [31]. For detecting nonmedical use of prescription drugs, sensitivity was lower (ranging from 0.61 to 0.66) but still comparable to other screening instruments [32], and specificity was high (0.97–0.98). Based on the results of this validation study, the TAPS Tool is among the instruments recommended by the National Institute on Drug Abuse and by SAMHSA to screen for opioid and other substance use in medical settings [10, 33].

While the primary focus of the TAPS Tool study was to evaluate the accuracy of the TAPS for identifying problem use and substance use disorders, data was also collected on operational characteristics (time and assistance required) and patient attitudes toward the TAPS Tool, for the purpose of informing its future integration into primary care practice. This study presents the planned secondary outcomes analysis of these data. The aims of our analysis were to determine the feasibility and acceptability of myTAPS among primary care patients, including in specific subpopulations that may have greater difficulty using an electronic self-administered screening tool.

We were interested in how our two outcomes (feasibility and acceptability) may differ among subpopulations of patients who may have greater difficulty completing an electronic self-administered instrument. We examined the hypothesis that myTAPS, in comparison to the interviewer format, would be less feasible and acceptable for individuals who have greater difficulty completing an electronic screener, and may thus appreciate the assistance that an interviewer can provide. Based on the existing literature on electronic substance use screening, we hypothesized that individuals from the following groups may have greater difficulty completing the myTAPS format, and would find the interviewer format more acceptable: males; older participants (age > 65 years); Hispanic participants; and individuals with less than high school education [21, 26, 28, 34–38.] Conversely, we hypothesized that the myTAPS format would be more feasible and acceptable, in comparison to the interviewer format, for those with current alcohol or drug use, females, racial minorities (African American), and younger participants (age 18–25 years), because self-administered screeners are typically preferred by individuals who are reporting stigmatized behavior or are from groups who suffer from high levels of substance use-associated stigma [39–44]. An electronic screening instrument was also hypothesized to be preferable for younger participants, who are highly acclimated to this technology. We further examined whether feasibility and acceptability differed based on the order in which the TAPS was administered, with a hypothesis that the first format received (whether it was myTAPS or interviewer-administered format) would be less feasible and acceptable than the second format, because participants would be more familiar with the TAPS Tool items on the second administration.

## Methods

The methods of the parent validation study (Clinical Trial registration: ClinicalTrials.gov identifier NCT02110693) are fully detailed in two preceding manuscripts [30, 31], and summarized here. The TAPS tool items are presented in a prior publication [31] and the instrument is available on the NIDA website (https://www.drugabuse.gov/taps/#/) [45].

### Participants and Recruitment

In a study of the National Drug Abuse Treatment Clinical Trials Network, between August 2014 and April 2015, 2000 participants, from five primary care clinics located in urban and suburban areas in the Eastern U.S., completed the TAPS Tool validation study. Eligible individuals were adults (18 years or older) who were current patients of one of the participating clinics. Individuals were excluded if they could not understand spoken English or were physically unable to use a tablet computer. Research assistants (RAs) consecutively approached patients in the waiting room to invite them to participate, and obtained verbal informed consent. Institutional review boards of the sites involved in the study (blinded for review) approved all study procedures.

### Study procedures

Participants completed the TAPS tool and other study assessments in a private room, and were informed that answers were confidential. All participants completed the TAPS Tool in both an interviewer-administered (administered by the RA) and an electronic self-administered (myTAPS) format. Each participant was randomly assigned to have the TAPS administered in one of two sequential orders (either interviewer-administered first followed by myTAPS, or myTAPS first followed by the interviewer-administered format). The myTAPS was delivered on a tablet computer (iPad), and participants had the option of hearing the question and response options read verbatim by a recorded female voice. Before starting myTAPS, participants were given the option of viewing, on the tablet, a brief tutorial on how to operate the electronic screening tool, include use of the touchscreen buttons to select responses and advance from one item to the next. The RA noted any participant requests for assistance, and recorded the reason(s) for the request, for both formats. The time required for completing myTAPS was recorded by the computer, and for the interviewer-administered format it was recorded in 1-min increments by the RA, using a stopwatch. Following completion of both formats of the TAPS, the RA verbally administered a brief survey. The survey was developed by the investigators to assess participant views on the tool’s feasibility and acceptability. Its items were informed by prior work on patient attitudes toward substance use screening [29].

#### Outcomes and measure

We assessed two types of outcomes: (1) feasibility (ease of use); and (2) acceptability (preference for the electronic versus interviewer-administered format). Survey responses used a 5-point Likert Scale (with options ranging from strongly agree to strongly disagree).

Feasibility of the TAPS tool was assessed by two self-reported questions from the survey, as well as RA-observed requests for assistance and the time (in minutes) required to complete the TAPS Tool. The survey questions addressed (1) the ease of use of the myTAPS tablet (“The iPad touch screen was easy to use”) and (2) the usefulness of the voice recording on the myTAPS (“The voice recording was helpful”). The participant’s response to the voice recording question was entered as ‘not applicable’ for those who did not use the voice recording; this allowed us to additionally use the response to this item as a measure of the number of participants who used the voice recording option. For those who requested assistance, the RAs recorded the reason(s); these reasons were further categorized into three categories: comprehension (e.g. problems understanding the meaning of a TAPS Tool item), difficulty using the tablet (e.g. trouble using the touchscreen), or technical issue(s) (e.g. tablet not operating correctly). Acceptability was assessed with two survey items: “I would prefer that a person asked me these questions in the doctor’s office instead of answering them myself on the iPad”; and “I would prefer answering these questions on an iPad instead of having a person ask me.”

### Statistical analyses

Demographic characteristics of participants and the three outcomes were summarized, for continuous variables, by their mean, median, and standard deviation (SD), and for categorical variables by their frequencies and percentages. To assess acceptability, survey collected measures were collapsed into three categories (1—strongly agree/agree, 2—neither agree nor disagree and 3—strongly disagree/disagree). Chi squared statistics were used to test whether the distribution of outcomes differed between subpopulations. For example, we tested whether the distribution of format preference differed between younger (18–25 years) and older (> 25 years) participants. Instead of assessing age as a single ordinal variable, we used age to examine whether older participants (> 65 years old) would prefer the interviewer-administered format because of greater difficulty using technology. Two logistic regression analyses were run with the dependent outcome variable dichotomized into agree (‘strongly agree’ and ‘agree’) versus disagree (‘neither agree nor disagree’, ‘disagree,’ and ‘strongly disagree’). The regression analysis assessed the association of each categorical outcome with each of the subpopulations; adjusted odds-ratios, 95% confidence intervals and p-values were obtained.

The feasibility measure ‘assistance requested’ was summarized by the number and proportion of participants who asked for assistance at least once while completing the myTAPS and the interviewed-administered format. The time to complete the two formats was summarized descriptively. The Chi squared test was used to evaluate ‘assistance requested’ to compare the subpopulation distributions. The Wilcoxon rank-sum test was used to evaluate time required to complete the TAPS tool for each subpopulation. Logistic regression was applied in the same manner as described above. Finally, we conducted analyses to determine if the order in which the two TAPS formats were administered affected feasibility. For these analyses, we compared the odds of requesting assistance and the time needed to complete the TAPS for those who received myTAPS first versus those who received the interviewed-administered format first. Format preference was measured through two separate items in the survey; one asked if the myTAPS format was preferred, and the other asked if the interviewer format was preferred. Among all participants, 69.8% (n = 1395) gave concordant answers to these two items. Concordance (i.e. those who preferred the myTAPS format did not prefer the interviewer format), is required in order to interpret results. Therefore, only participants with concordant responses were included in the analysis of format preference. For these analyses, illicit and nonmedical prescription drug uses were combined into a single variable, to maximize the available sample for the planned analyses. All regression models included age, education level, race, ethnicity, sex, and substance use (alcohol, drug, and nonmedical prescription drug use). Analyses were performed using STATA 14 software (StataCorp. 2015. Stata Statistical Software: Release 14. College Station, TX: StataCorp LP).

## Results

Participant characteristics are summarized in Table 1.

**Table 1:** Demographic characteristics of the 2000 participants

Characteristic	Participants N (%)
Gender	
Women	1124 (56.2)
Men	874 (43.7)
Other^a^/refused	2 (1.0)
Age	
Mean (years), standard deviation	46.0, SD = 14.7
Younger participants: 18–25 years old	225 (11.3)
Older participants: > 65 years old	160 (8.0)
Race	
White	667 (33.4)
Black/African American	1112 (55.6)
Multiracial	66 (3.3)
Other^b^	148 (7.4)
Refused	7 (0.4)
Ethnicity	
Hispanic	233 (11.7)
Non-Hispanic	1761 (88.1)
Other^c^/refused	6 (0.3)
Education level	
Less than high school degree (HS)	383 (19.2)
HS or GED	578 (28.9)
Higher than HS (some college, associate, bachelor or graduate degree)	1038 (51.9)
Other^a^	1 (0.1)
Unhealthy substance use (reported on myTAPS 1^d^)^e^	
Tobacco	906 (45.3)
Alcohol	858 (42.9)
Illicit drug	492 (24.6)
Prescription drug	245 (12.3)

^a^‘Don’t know’ (n = 1)

^b^Asian (n = 35), American-Indian or Alaska Native (n = 12), unknown (n = 32), other not specified (n = 69)

^c^‘Don’t know’ (n = 5)

^d^Electronic self-administered format of the Tobacco, Alcohol, Prescription medication, and other Substance use, screening step

^e^Substance use categories are not mutually exclusive

A majority of participants (56.2%) were women, and the mean age was 46 years (SD = 14.7); 11.3% were 18–25 years old, and 8.0% were over 65. Just over half (55.6%) of the participants were Black/African American and 11.7% were Hispanic. Nineteen percent had less than high school education. Based on responses to the myTAPS (TAPS-1 items), 42.9% had unhealthy alcohol use, 24.6% used illicit drugs, and 12.3% had nonmedical use of prescription drugs, in the past year. There was some overlap between illicit and nonmedical prescription drug use, with 91 participants (4.6% of the sample) screening positive for both.

### Feasibility

Throughout the results section, results of the adjusted analyses are presented in the text, while results of bivariate analyses can be found in the specified tables.

### Self-reported feasibility (Table 2)

The measures of feasibility showed that most participants (98.3%) found the tablet (myTAPS) easy to use. In the multivariate analysis, women had two times the odds of reporting that the tablet was easy to use, in comparison to men (OR = 2.09, 95% CI 1.01–4.33). Those who screened positive for prescription drug use had lower odds of reporting that the tablet was easy to use (OR = 0.36, 95% CI 0.15–0.83).

**Table 2:** Self-reported feasibility of the myTAPS (N = 2000)

	Number of participants n (%)	Tablet easy to use			Audio guidance used		
		Agree^a^ n (%)	Pearson’s Chi squared test	Logistic regression adjusted odds ratio^b^ [95% confidence interval]	Yes n (%)	Pearson’s Chi squared test	Logistic regression adjusted odds ratio^b^ [95% confidence interval]
All participants	2000 (100)	1965 (98.3)			365 (18.3)		
Gender							
Women	1124 (56.2)	1111 (98.8)	4.55*	2.09 [1.01; 4.33]*	149 (13.3)	42.89***	0.52 [0.41; 0.66]***
Men or other	876 (43.8)	854 (97.6)			216 (24.7)		
Age							
Younger participants (18–25)	225 (11.3)	221 (98.2)	0.01	0.68 [0.23; 2.05]	12 (5.3)	28.35***	0.30 [0.16; 0.54]***
Participants (> 25)	1775 (88.7)	1744 (98.3)			353 (19.9)		
Older participants (> 65)	160 (8.0)	158 (98.8)	10.24**	1.40 [0.33; 6.04]	47 (29.4)	14.43***	1.79 [1.22; 2.61]**
Participants < 65	1840 (92.0)	1807 (98.3)			318 (17.3)		
Race							
Black/African American	1112 (55.6)	1092 (98.2)	0.14	0.92 [0.45; 1.90]	221 (19.9)	4.43*	1.30 [1.01; 1.68]*
Non-Black/African American	888 (44.4)	873 (98.4)			144 (16.2)		
Ethnicity							
Hispanic	233 (11.7)	229 (98.3)	0.0004	1.00 [0.33; 3.03]	61 (26.2)	11.11**	1.92 [1.36; 2.74]***
Non-Hispanic	1767 (88.3)	1736 (98.3)			304 (17.2)		
Education level							
Less than high school (HS)	383 (19.2)	375 (97.9)	0.43	0.84 [0.37; 1.91]	115 (30.0)	44.03***	2.01 [1.54; 2.63]***
HS level or higher	1617 (80.8)	1590 (98.4)			250 (15.5)		
Alcohol use							
Screened positive^c^	858 (42.9)	846 (98.6)	0.82	1.78 [0.83; 3.86]	154 (18.0)	0.09	0.99 [0.77; 1.28]
Screened negative	1142 (57.1)	1119 (98.1)			211 (18.5)		
Illicit drug use							
Screened positive^c^	492 (24.6)	482 (98.0)	0.43	0.97 [0.42; 2.23]	93 (18.9)	0.19	0.92 [0.69; 1.24]
Screened negative	1508 (75.4)	1483 (98.4)			272 (18.0)		
Prescription drug use							
Screened positive^c^	245 (12.3)	236 (96.3	6.50*	0.36 [0.15; 0.83]*	53 (21.6)	2.14	1.10 [0.77; 1.57]
Screened negative	1755 (87.7)	1729 (98.6)			312 (17.8)		
Order of administration							
myTAPS^d^ first (= ref)	1002 (50.1)	985 (98.4)	0.13	0.82 [0.41; 1.64]	188 (18.8)	0.35	0.97 [0.76; 1.23]
Interviewer-administered first	998 (49.9)	980 (98.20)			177 (17.7)		

^a^The frequency of answers “agree/strongly agree” to the item of the survey

^b^Logistic regression models controlled for all covariates included in Table 2 (age, education level, race, ethnicity, sex, substance use (alcohol, drug, and prescription drug use)

^c^Screened positive for unhealthy use on myTAPS-1

^d^Electronic self-administered format of the tobacco, alcohol, prescription medication, and other substance use (TAPS)

*p value < 0.05, **p-value < 0.01, *** p-value < 0.001

The myTAPS audio guidance was used by a minority (18.3%) of participants. Participants over age 65 more frequently reported use of the audio guidance (29.4% versus 17.3%, OR = 1.79, 95% CI 1.22–2.61), as did those who were Hispanic (26.2% versus 17.2%, OR = 1.92, 95% CI 1.36–2.74), those who were Black/African-American (19.9% versus 16.2%, OR = 1.30, 95% CI 1.01–1.68), and those who had less education (30% versus 15.5%, OR = 2.01, 95% CI 1.54–2.63). Conversely, the audio guidance was used less by women (OR = 0.52, 95% CI 0.41–0.66), and by young participants (OR = 0.30, 95% CI 0.16–0.54).

### Requests for assistance (Table 3)

One-quarter (25.0%) of the study population (n = 500) requested assistance with myTAPS, while 8.1% (n = 162) requested assistance with the interviewed-administered format. Participants most frequently requested assistance with myTAPS because of difficulty using the tablet (7.8% of the sample, n = 155), followed by comprehension problems (6.9%, n = 137), and technical issues (6.5%, n = 129). An additional 75 (3.8%) requested assistance for more than one reason, and for 4 participants the reason for requesting assistance was not recorded.

**Table 3:** Assistance requested during the completion of the myTAPS Tool (N = 2000)

	Number of participant n (%)	Assistance requested^a^ n (%)	Pearson’s Chi square test	Logistic regression adjusted^b^ odds ratio [95% confidence interval]
All participants	2000 (100)	500 (25.0)		
Gender				
Women	1124 (56.2)	262 (23.3)	3.91*	0.89 [0.71; 1.10]
Men or other	876 (43.8)	238 (27.2)		
Age				
Younger participants (18–25)	225 (11.3)	34 (15.1)	13.22***	0.62 [0.42; 0.91]*
Participants > 25	1775 (88.7)	466 (26.3)		
Older participants (> 65)	160 (8.0)	77 (48.1)	49.6***	2.79 [1.98; 3.93]***
Participants < 65	1840 (92.0)	423 (23.0)		
Race				
Black/African American	1112 (55.6)	280 (25.2)	0.04	0.94 [0.75; 1.17]
Non-Black/African American	888 (44.4)	220 (24.8)		
Ethnicity				
Hispanic	233 (11.7)	62 (26.7)	0.36	1.05 [0.75; 1.47]
Non-Hispanic	1767 (88.3)	438 (24.8)		
Education level				
Less than high school (HS)	383 (19.2)	146 (38.1)	43.49***	2.08 [1.62; 2.67]***
HS or higher	1617 (80.8)	354 (21.9)		
Alcohol use				
Screened positive^c^	858 (42.9)	196 (22.9)	3.73	0.89 [0.69; 1.08]
Screened negative	1142 (57.1)	304 (26.6)		
Illegal drug use				
Screened positive^c^	492 (24.6)	115 (23.4)	0.92	0.90 [0.69; 1.17]
Screened negative	1508 (75.4)	385 (25.5)		
Prescription drug use				
Screened positive^c^	245 (12.3)	75 (30.6)	4.69*	1.37 [1.00; 1.88]
Screened negative	1755 (87.7)	425 (24.2)		
Order of administration				
myTAPS first^d^ (= ref)	1002 (50.1)	278 (27.7)	8.07**	1.35 [1.09; 1.67]*
Interviewer-administered first	998 (49.9)	222 (22.2)		

^a^Assistance was requested one or more times

^b^Logistic regression models controlled for all covariates include in Table 5a (age, education level, race, ethnicity, sex, substance use (alcohol, drug, and prescription drug use, and order of administration)

^c^Screened positive for unhealthy use on myTAPS-1

^d^Electronic self-administered format of the tobacco, alcohol, prescription medication, and other substance use (TAPS)

*p-value < 0.05, **p-value < 0.01, ***p-value < 0.001

Examining results by subpopulation, participants over 65 years old requested assistance with myTAPS at twice the rate of younger participants (48.1% versus 23.0%, OR = 2.79, 95% CI 1.98–3.93). Assistance was also more frequently requested by participants having less than high school education, in comparison with those having high school or higher level education (38.1% versus 21.9%, OR = 2.08, 95% CI 1.62–2.67). Participants more frequently requested assistance on the first format they completed, regardless of whether it was the myTAPS (27.7% versus 22.2%, OR = 1.35, 95% CI 1.09–1.67) or the interviewer format (11.5% versus 4.7%, OR = 3.17, 95% CI 2.20–4.57).

### Time to complete the TAPS tool (Table 4)

Across all participants, the median time to complete myTAPS was 4.00 min (SD = 2.57, range 1–27 min), and it was completed by 90% of participants in 7 min or less. By comparison, the interviewer format had a median completion time of 2.00 min (SD = 1.00, range < 1 to 11 min), and was completed by 90% of participants in 3 min or less. Based on results of the Wilcoxon test, the time to complete myTAPS was higher for participants who were older (median = 5.00, mean = 6.14, SD = 3.30) Black/African American (median = 4.00, mean = 4.73, SD = 2.58), Hispanic (median = 4.00, mean = 5.03, SD = 3.00), had lower education (median = 5.00, mean = 6.00, SD = 3.26), or screened positive for illicit (median = 4.00, mean = 5.31, SD = 2.52) or prescription drug use (median = 5.00, mean = 4.93, SD = 2.72), in comparison to participants without these characteristics. Following a similar pattern to requests for assistance, more time was required to complete the format of the TAPS tool that was received first, whether it was the myTAPS (median = 4.00, mean = 4.85, SD = 2.84) or the interviewer-administered (median = 4.00, mean = 4.11, SD = 2.21) version. Less time was needed by women (median = 3.00, mean = 3.95, SD = 2.00), and by younger participants (median = 3.00, mean = 3.00, SD = 1.08) to complete myTAPS.

**Table 4:** Time to complete the myTAPS (N = 2000)

	Number of participants n (%)	Time in minutes median/mean (SD^a^)	Wilcoxon test^b^
All participants	2000 (100)	4.00/4.48 (2.57)	
Gender			
Women	1124 (56.2)	3.00/3.95 (2.00)	10.99***
Men or other	876 (43.8)	4.00/5.17 (2.94)	
Age			
Younger participants (18–25)	225 (11.3)	3.00/3.00 (1.08)	11.20***
Participants > 25	1775 (88.7)	4.00/4.67 (2.67)	
Older participants (> 65)	160 (8.0)	5.00/6.14 (3.30)	− 9.03***
Participants < 65	1840 (92.0)	4.00/4.34 (2.44)	
Race			
Black/African American	1112 (55.6)	4.00/4.73 (2.58)	− 6.48***
Non-Black/African American	888 (44.4)	3.00/4.17 (2.52)	
Ethnicity			
Hispanic	233 (11.7)	4.00/5.03 (3.00)	− 3.52***
Non-Hispanic	1767 (88.3)	4.00/4.41 (2.50)	
Education level			
Less than high school (HS)	383 (19.2)	5.00/6.00 (3.26)	− 12.28***
HS or higher	1617 (80.8)	4.00/4.12 (2.23)	
Alcohol use			
Screened positive^c^	858 (42.9)	4.00/4.61 (2.68)	− 1.87
Screened negative	1142 (57.1)	4.00/4.38 (2.48)	
Illegal drug use			
Screened positive^c^	492 (24.6)	4.00/4.93 (2.52)	− 5.91***
Screened negative	1508 (75.4)	4.00/4.34 (2.57)	
Prescription drug use			
Screened positive^c^	245 (12.3)	5.00/5.31 (2.72)	− 6.33***
Screened negative	1755 (87.7)	4.00/4.37 (2.53)	
Order of administration			
myTAPS first	1002 (50.1)	4.00/4.85 (2.84)	6.59***
Interviewer-administered first	998 (49.9)	4.00/4.11 (2.21)	

Electronic self-administered format of the tobacco, alcohol, prescription medication, and other substance use (TAPS)

^a^Standard deviation

^b^Wilcoxon test assessed the difference between each subgroup versus all other participants (i.e. women versus non-women)

^c^Screened positive on myTAPS-1

*p-value < 0.05, **p-value < 0.01, ***p-value < 0.001

### Acceptability (Table 5)

A majority (52.7%) of participants had no preference regarding the myTAPS versus the interviewer-administered format of the TAPS Tool. Thee myTAPS format was preferred by 27.5% (n = 383) of participants, while the interviewer-administered format was preferred by 19.9% (n = 277). Participants with less than a high school education had almost three times greater odds of preferring the interviewer-administered format (OR = 2.75, 95% CI 2.00–3.78**)**. In comparison to men, women were somewhat more likely to prefer myTAPS (OR = 1.29, 95% CI 1.00–1.66). Those who screened positive for drug use were also more likely to prefer the myTAPS over the interviewer-administered format (OR = 1.43, 95% CI 1.09–1.88).

**Table 5:** Format preference (N = 1395)

	Number of participants n (%)	myTAPS^a^ preferred			Interviewer-administered format preferred		
		Agree^b^ n (%)	Pearson’s Chi squared test	Logistic regression adjusted odds ratio^c^ [95% confidence interval]	Agree^b^ n (%)	Pearson’s Chi squared test	Logistic regression adjusted odds ratio^c^ [95% confidence interval]
All participants	1395 (100)	383 (27.5)			277 (19.9)		
Gender							
Women	805 (57.7)	233 (28.9)	2.12	1.29 [1.00; 1.66]*	145 (18.0)	4.07*	0.76 [0.58; 1.01]
Men or other	590 (42.3)	150 (25.4			132 (22.4)		
Age							
Younger participants (18–25)	174 (12.5)	41 (23.6)	1.51	0.68 [0.47; 1.00]	37 (21.3)	0.25	1.25 [0.83; 1.88]
Participants > 25	1221 (87.5)	342 (28.0)			240 (19.7)		
Older participants (> 65)	109 (7.8)	20 (18.4)	4.92*	0.62 [0.37; 1.04]	26 (23.9)	1.19	1.15 [0.75; 1.72]
Participants < 65	1286 (92.2)	363 (28.2)			251 (19.5)		
Race							
Black/African American	734 (52.6)	210 (28.6)	1.04	1.12 [0.88; 1.43]	140 (19.1)	3.03	0.77 [0.58; 1.02]
Non-Black/African American	661 (47.4)	173 (26.2)			137 (20.7)		
Ethnicity							
Hispanic	154 (11.0)	37 (24.0)	1.02	0.89 [0.60; 1.34]	38 (24.7)	2.53	1.14 [0.75; 1.73]
Non-Hispanic	1241 (89.0)	346 (27.9)			239 (19.3)		
Education level							
Less than high school (HS)	229 (16.4)	43 (18.8)	10.36**	0.56 [0.39; 0.80]**	81 (35.4)	41.44***	2.75 [2.00; 3.78]***
HS level or higher	1166 (83.6)	340 (29.2)			196 (16.8)		
Alcohol use							
Screened positive^d^	596 (42.7)	174 (29.2)	1.58	1.07 [0.83; 1.38]	112 (18.8)	0.74	0.87 [0.65; 1.16]
Screened negative	799 (57.3)	209 (26.2)			165 (20.7)		
Any drug use^e^							
Screened positive^d^	415 (29.8)	133 (32.1)	6.26*	1.43 [1.09; 1.88]**	83 (20.0)	0.01	0.98 [0.72; 1.34]
Screened negative	980 (70.3)	250 (25.5)			194 (19.8)		

Included in this analysis were individuals who gave concordant answers regarding format preference; 605 individuals with non-concordant answers were excluded

^a^Electronic self-administered format of the tobacco, alcohol, prescription medication, and other substance use (TAPS)

^b^Includes responses of “agree” and “strongly agree”

^c^Logistic regression models controlled for all covariates included in Table 4 (age, education level, race, ethnicity, sex, substance use (alcohol, and any drug use)

^d^Screened positive for unhealthy use on myTAPS-1

^e^Illicit drug and/or non-medical prescription drug use

*p-value < 0.05, **p-value < 0.01, *** pvalue < 0.001

## Discussion

The myTAPS Tool, which efficiently screens and assesses tobacco, alcohol, and illicit or nonmedical prescription drug use (including opioids), was feasible and acceptable for the majority of participants in this diverse sample of adult primary care patients. While we detected small differences among some subpopulations who may be expected to have difficulty with an electronic self-administered screener, the overarching finding of our analysis was that most patients would be able and willing to complete screening with the myTAPS tool.

The myTAPS required more time to complete than the interviewer format, but as a self-administered screener, (which could be completed in the waiting room or prior to the clinic visit), requiring a median time of 4.0 min, myTAPS would be feasible in most primary care settings. Overall, one-quarter of participants requested some assistance with myTAPS. This is comparable to the rate of assistance found in a study of electronic self-administered formats of the Single-Item Screening Questions for alcohol and drugs, in which 29% of participants needed assistance [21], but higher than what has been reported for some other electronic self-administered screening tools. In a prior study of adult patients enrolled from public primary care clinics, 11% needed assistance with the Substance Use Brief Screen (SUBS) [32], and 5.3% needed assistance with the more complex ACASI-ASSIST [23]. Participants with lower education, as well as those over 65 years old, more frequently requested assistance and required more time to complete myTAPS screening. Prior studies have similarly shown that electronic and self-administered questionnaires may be more difficult for primary care patients having less computer familiarity or lower literacy [26, 28]. Our study thus indicates that while the majority of primary care patients can complete the myTAPS without assistance, clinical settings serving primarily older and less educated patients should have an interviewer-administered screening approach available, and all settings should be prepared to offer assistance to some individuals. Given that most patients would be able to complete the myTAPS on their own, adoption of a predominantly electronic screening approach may free up the time for clinical staff to help those patients who need it.

The use of audio guidance during the completion of myTAPS screening was frequent (18%), and significantly higher among participants with lower education, age > 65 years, and participants who were Hispanic or Black/African American. The higher use of audio guidance by these participants could potentially be explained by their lack of confidence in using new technology, or by low literacy [21, 26, 28]. Prior studies have also found that patients who are non-native English speakers could have difficulty understanding electronic self-administered screening questions [34, 36, 46]. Because our study did not collect data on primary language, we were unable to assess whether language was the reason that members of racial and ethnic minorities in our sample reported more use of the audio guidance.

Interestingly, we found that participants who screened positive for nonmedical use of prescription drugs requested more assistance, and required more time to complete screening in both formats of the TAPS. Our previous studies found that primary care patients had difficulty understanding screening items about prescription medications [29, 47]. Confusion about how to report nonmedical prescription drug use could underlie the lower feasibility among individuals who reported prescription drug use in our sample. In settings where nonmedical use of prescription medications is of particular concern, practices may consider adding an introduction to the screening questions, similar to that used for the ACASI ASSIST:^1^ [23].

Regarding acceptability, most participants (52.7%) had no preference regarding electronic versus interviewer-administered screening. The interviewer-administered format was preferred by those having lower levels of education, perhaps because of the feasibility issues discussed above. Women and individuals who screened positive for drug use preferred the myTAPS format. Generally, self-administered questionnaires are preferred and are more accurate when asking about a stigmatized behavior [16, 48]. This finding is consistent with prior studies reporting that individuals with substance use, and especially women who use drugs, feel highly stigmatized [29, 40, 49–52], which could cause them to be less comfortable answering screening questions face-to-face.

### Limitations

Our study has some limitations. While it is not surprising that individuals who reported substance use required more time to complete the TAPS (since they received more questions), for all participants the time required to complete the TAPS Tool was likely overestimated. Time was measured as the time to complete both the TAPS-1 and TAPS-2, and the recommended skip pattern was not followed because the validation study sought to study the performance of the TAPS-1 and TAPS-2 both separately and in combination. The time required to complete the TAPS Tool that we reported here should thus be viewed as the maximum time for patients to complete screening. Nonetheless, we found that the time was brief and would likely be feasible in a primary care setting (myTAPS: median time 4.0 min, and interviewer-administered format: median time 2.0 min). In comparison, the time to complete the longer ACASI-ASSIST was found in a prior study to be 3 to 5 min, [23, 29] while the interviewer-ASSIST required 5–15 min [53, 54].

Our survey regarding acceptability of the TAPS tool was not validated, and richer information about patient attitudes and preferences might have been gained through qualitative interviews. Having the RA administer the survey could have introduced social desirability bias. As detailed in the Methods section, there was inconsistency of responses regarding preference for interviewer-administered format versus myTAPS that required us to exclude 30% of the sample from the format preference analysis. Nevertheless, the survey gave consistent results on related items assessed and its findings were consistent with prior literature [16, 26, 28, 48]. Although the study was conducted in a general adult primary care population, the prevalence of substance use in our sample was somewhat higher than may be found in other primary care settings [55]. The study sites were only in urban and suburban areas, which may limit the generalizability of our findings to other populations, including those living in rural areas. Acceptability and feasibility may differ depending on characteristics of the population and the setting in which screening is being conducted. Finally, because the TAPS Tool was only available in English at the time of our study, we are unable to evaluate its feasibility and acceptability in other languages. A Spanish version has since been developed and pilot tested [56].

## Conclusions

The myTAPS Tool would be feasible in most primary care clinical settings, and was well accepted by patients in this large and diverse sample of adult primary care patients. However, primary care practices that choose this format should be prepared to offer assistance to some patients, particularly those who are older or have less than a high school level of education, and should have the capacity to screen using an interviewer-administered approach when required. Future studies should assess the acceptability and the feasibility of myTAPS screening when it is implemented in routine practice.

## Data Availability

The datasets used and/or analysed during the current study are available from the corresponding author or from the NIDA Center for the Clinical Trials Network on reasonable request.

## Abbreviations

TAPS: tobacco, alcohol, prescription medication and other substances use screening tool
myTAPS: electronic self-administered format TAPS
SD: standard deviation
TAPS-1: first step of the TAPS tool (4 screening-items)
TAPS-2: second step of the TAPS tool (2–3 assessment items for each substance)

## Footnotes

1 “*Some of the substances listed may be prescribed by a doctor (like stimulants, sedatives, and pain medications). For this interview, please do not report medications that are used as prescribed by your doctor. However, please do report use of these medications if you have taken them 'recreationally'*—*which means taking medications that were prescribed to you or to someone else just for the feeling or experience they cause, to get high, or taking them more often or at* higher doses *than prescribed.”*
